# Effects of Dietary *Pediococcus acidilactici* and Isomaltooligosaccharide on Growth Performance, Immunity, and Antioxidant Defense in Juvenile Common Carp

**DOI:** 10.1155/2023/1808640

**Published:** 2023-02-14

**Authors:** Milad Maniat, Amir Parviz Salati, Nasim Zanguee, Seyed Mohammad Mousavi, Seyed Hossein Hoseinifar

**Affiliations:** ^1^Department of Fisheries, Faculty of Marine Natural Resources, Khorramshahr University of Marine Science and Technology, Khorramshahr, Iran; ^2^Department of Fisheries, Faculty of Natural Resources, Gorgan University of Agricultural Science and Natural Resources, Gorgan, Iran

## Abstract

The aim of this study was to investigate the synbiotic effects of *Pediococcus acidilactici* (PA) and isomaltooligosaccharide (IMO) on the performance of juvenile common carp (*Cyprinus carpio*). A total of 360 fish (17.22 ± 0.19 g) were randomly divided into six groups with three replicates of 20 fish each. The trial continued for 8 weeks. The control group was fed only basal diet; PA was fed basal diet supplemented with 1 g/kg (10^10^ CFU/kg) PA, IMO5 (5 g/kg IMO), IMO10 (10 g/kg IMO), PA-IMO5 (1 g/kg PA and 5 g/kg IMO), and PA-IMO10 (1 g/kg PA and 10 g/kg IMO). The results indicated that the diet containing 1 g/kg PA and 5 g/kg IMO significantly increased the fish growth performance and decreased the feed conversion ratio (*p* < 0.05). Overall, blood biochemical parameters, serum (lysozyme, complements C3 and C4) and mucosal (protein, total immunoglobulin, and lysozyme) immune responses, and antioxidant defense of fish also improved in the PA-IMO5 group (*p* < 0.05). Therefore, a combination of 1 g/kg (10^10^ CFU/kg) PA and 5 g/kg IMO can be recommended as a beneficial synbiotic additive and immunostimulant in juvenile common carp.

## 1. Introduction

In recent years, the culture of common carp has developed significantly. Common carp is one of the most significant species of warm-water fish, which accounts for 8.6% of the total production of farmed fish [1]. The production of common carp has increased from 3.42 million tons in 2012 to 4.24 million tons in 2020, which shows the economic importance of this species [1]. Moreover, common carp is one of the most important freshwater fish species that is widely used in scientific studies. Due to the development of intensive fish farming systems, antibiotics and different kinds of antimicrobial agents are widely used in aquaculture in order to prevent diseases and improve the growth performance, feed efficiency, immune system status, and survival rate of farmed fish [2]. However, antibiotics have adverse effects; they can promote microbial antibiotic resistance and cause intestinal microbial flora imbalance; furthermore, they can harm the environment and upset the balance of environmental microflora when excreted from farms and dispersed into waterways, leading to unwanted side effects on aquatic organism health [3]. Nowadays, using dietary supplements such as probiotics, prebiotics, and synbiotics is recommended to enhance the growth and health of fish species [4–7].

*Pediococcus acidilactici* (PA) is a nonpathogenic and gram-positive coccus which is a facultative anaerobic bacterium. It can grow in a wide range of environmental conditions including temperature, pH, and osmotic pressure. The rapid multiplication of this bacterium in the gastrointestinal tract causes a competitive elimination of pathogens and reduces the risk of diseases. Oral administration of this bacterium as a probiotic significantly reduces mortality from infectious diseases and increases the survival rate [8]. Dietary supplementation of *P. acidilactici* balances the population of intestinal bacterial flora, and it also stimulates a nonspecific immune response [9]. A variety of bacteriocins are secreted by different strains of *P. acidilactici* [10, 11] which increase fish resistance to both gram-positive and gram-negative pathogenic bacteria [9]. Prebiotics are nondigestible food ingredients which improve host health by selectively stimulating the growth and activity of some beneficial intestinal bacteria [12]. Romano et al. [13] reported that a combination of dietary pregelatinized starch and isomaltooligosaccharide at 0.5% improved feed efficiency and physiological status in juvenile African catfish (*Clarias gariepinus*). Moreover, Li et al. [14] confirmed the synergistic effects of dietary *Bacillus* as a probiotic isomaltooligosaccharide in shrimp.

It has been proven that the combination of prebiotics and probiotics—known as synbiotics—can lead to higher survival and growth rates of probiotic bacteria by creating synergistic effects [15]. Modanloo et al. [6] reported a positive impact of supplementation of a common carp diet with 10 g/kg galactooligosaccharide and 1 g/kg *P. acidilactici* on some mucosal or serum immune responses. Based on the results of Miao et al. [16], *P. acidilactici* supplementation (1 × 10^8^ CFU/g diet), single or combined with yeast (*Saccharomyces cerevisiae*) and/or *β*-glucan, can be recommended as a promising immunostimulant in prawn farming. Moreover, the combination of a low level of mannan oligosaccharides and *P. acidilactici* as a synbiotic additive can be an effective tool to potentiate European sea bass (*Dicentrarchus labrax*) juveniles' growth and disease resistance when supplemented in low fishmeal and fish oil diets [7]. Furthermore, the combined effects of sodium alginate (5 or 10 g/kg) with *P. acidilactici* (0.9 × 10^7^ CFU/g) caused more evident immune responses compared to the control and singular administration in Asian sea bass (*Lates calcarifer*) juveniles [17]. Therefore, the effects of dietary *P. acidilactici* as a probiotic and prebiotic isomaltooligosaccharide on growth, blood biochemical parameters, serum and mucosal immune responses, and antioxidant defense in juvenile common carp were investigated in our study.

## 2. Material and Methods

### 2.1. Experimental Diets

*Pediococcus acidilactici* (PA, lyophilized form, 1 × 10^13^ CFU/kg) and isomaltooligosaccharide (IMO) were provided by Takgene Co. (Tehran, Iran) and Orafti Co. (Belgium), respectively. A commercially available common carp diet (Faradaneh Co., Shahrekord, Iran) was used as the basal diet [17, 18]. The proximate composition of the commercial (basal) diet consisted of 35% crude protein, 6% crude lipid, 5% crude fiber, 9% ash, and 1.25% phosphorus. Briefly, the basal diet was well mixed with PA and/or IMO and the required amount of distilled water was added to the mixture. Then, the dough was passed through a meat grinder and the strings were dried at room temperature. The diets were packed into plastic bags and kept at 4°C until feeding.

### 2.2. Fish and Experimental Design

The experiment was conducted at Khorramshahr University of Marine Science and Technology (Khorramshahr, Iran) in accordance with the ethics and animal care committee of this university. Juvenile common carp (*Cyprinus carpio*) were obtained from a local fish farm (Khorramshahr, Iran) and transported to the Fisheries Wet Laboratory. After being disinfected with sodium chloride (2% for 15 min), the fish were transferred to two 2000 l circular tanks and kept for two weeks in order to acclimatize. The fish were fed with the aforementioned commercial (basal) diet twice a day (09:00 and 16:00 h) to apparent satiation. After acclimatization, a total of 360 fish with an initial weight of 17.22 ± 0.19 g were divided into eighteen 300 l fiberglass circular tanks (20 fish per tank) in a completely randomized design consisting of six treatments. Each treatment was performed in 3 replications. In order to prepare the experimental diets, PA and IMO were added to the aforementioned commercial (basal) diet as follows: control (diet) group (0 g/kg PA and 0 g/kg IMO), PA (1 g/kg (10^10^ CFU/kg) PA), IMO5 (5 g/kg IMO), IMO10 (10 g/kg IMO), PA-IMO5 (1 g/kg PA and 5 g/kg IMO), and PA-IMO10 (1 g/kg PA and 10 g/kg IMO).

Fish were hand-fed the experimental diets for 8 weeks twice a day (09:00 and 16:00 h) to apparent satiation. Any uneaten food was collected 30 min after each meal, dried at 70°C, and then weighed to calculate the feed conversion ratio. All tanks were aerated using air stones connected to an air pump, and water exchange at a level of 30% of the total volume was performed daily. The light condition was 12 : 12 h (light/dark). A portable apparatus (Hach HQ40d, Loveland, Colorado, USA) was used to measure water temperature, dissolved oxygen, and pH. The water quality was monitored daily during the trial, and the ranges were as follows: temperature, 26.39 ± 0.68°C; dissolved oxygen, 7.78 ± 0.53 mg/l; and pH, 7.14 ± 0.21. No critical level was recorded for NH_3_.

### 2.3. Growth Performance

After 8 weeks of rearing, fish were starved for 24 h. Then, all fish were anesthetized using clove powder (200 mg/l) and weighed individually. The weight gain (WG), specific growth rate (SGR), feed conversion ratio (FCR), and survival rate were calculated for each replicate based on the standard formulas [19].

### 2.4. Blood and Mucus Sampling

At the end of the feeding trial, after 24 h of food deprivation, blood samples were randomly collected by nonheparinized syringes from the caudal vein of 3 fish per tank and centrifuged at 5000 × g for 10 min at 4°C [19]. The fish were anesthetized with clove powder (200 mg/l) prior to sampling. The serum was divided into two parts for antioxidant defense analysis and immunological tests and kept at −80°C until analysis. Also, fish mucus was collected as described by Ross et al. [20]. Briefly, 3 fish from each tank were put in a plastic bag containing 2 ml of 50 mM sodium chloride. Plastic bags containing fish were shaken for 2 min, and then the fish were removed from the bags. The collected mucus in the bag was centrifuged at 3000 × g for 10 min at 4°C, and then the supernatant was removed and stored at -80°C [21].

### 2.5. Biochemical and Immunological Analysis

Serum biochemical parameters including glucose, cholesterol, triglycerides, protein, albumin, high-density lipoproteins (HDL), and low-density lipoproteins (LDL) were analyzed by a biochemical autoanalyzer (Mindray, China) using Pars Azmoon commercial kits (Karaj, Iran) as described by the company.

Serum lysozyme activity was measured by the method recommended by Ellis [22] with some modifications. 200 *μ*l of 0.2 mg/ml suspension of *Micrococcus luteus* in 0.05 M sodium phosphate buffer (pH = 6.2) was mixed with 10 *μ*l of serum samples, and absorption of samples was read for 5 min consequently at 530 nm. PBS was used as blank. Each unit of enzyme activity was calculated as the amount of enzyme that reduces the absorption by 0.001 per min per ml of serum. Serum lysozyme activity was calculated in terms of *μ*g/ml. In order to measure the activity of C3 and C4, the commercial kits (Pars Azmoon, Iran) were used as described by the company.

Mucus total protein was measured by the method described by Lowry et al. [23]. Measurement of total immunoglobulin (total Ig) was performed based on measurement of the total protein content of mucus before and after precipitation of immunoglobulin using a 12% polyethylene glycol solution. The difference between the protein before and after precipitation was considered total immunoglobulin [24]. Lysozyme activity was also measured as described above.

### 2.6. Antioxidant Enzyme Analysis

Catalase (CAT, E.C. 1.11.1.6) activity was measured in serum by the decrease in absorbance at 240 nm (*e* = 40 M^−1^ cm^−1^) using 50 mM H_2_O_2_ as substrate [25]. Superoxide dismutase (SOD, E.C. 1.15.1.1) activity was measured at 550 nm as the degree of inhibition of cytochrome c reduction by O_2_^−^ generated by the xanthine oxidase/hypoxanthine system, according to McCord and Fridovich [26]. The activity of glutathione peroxidase (GPx) was evaluated with a GPx detection kit (Randox, Crumlin, UK) according to the manufacturer's instruction manual and Noguchi et al. [27]. The total amount of lipid peroxidation was assayed by the content of malondialdehyde (MDA) as described by Buege and Aust [28]. Briefly, one volume of serum was mixed with two volumes of a stock solution of 15% *w*/*v* trichloroacetic acid, 0.375% *w*/*v* thiobarbituric acid, and 0.25 M hydrochloric acid thoroughly. The solution was kept in a boiling water bath for 15 min. After cooling, the precipitate was removed by centrifugation at 1000 × g for 10 min. The absorbance of the clear supernatant was determined at 535 nm.

### 2.7. Statistical Analysis and Calculations

All data were expressed as mean ± standard error (mean ± SE). The normality of the data and homogeneity of variance were checked by the Shapiro-Wilk and Levene tests, respectively. One-way analysis of variance (ANOVA) was used to compare between treatments, and its significance was determined by the Duncan posttest (*p* < 0.05). The tests were performed in SPSS software version 23. The following parameters were also calculated: weight gain (%) = 100 × (final weight–initial weight)/initial weight; specific growth rate (SGR, %/day) = 100 × (ln final weight–ln initial weight)/day; feed conversion ratio (FCR, g/g) = dry feed intake/(final weight–initial weight); andsurvival rate (%) = 100 × final number of fish/initial number of fish.

## 3. Results

### 3.1. Growth Performance and Feed Utilization

The growth performance and nutrition indices of treatments are shown in Table 1. There were no significant differences in the fish survival rate among treatments at the end of the experiment (*p* > 0.05). Growth indices including the final weight, weight gain, and specific growth rate were significantly higher in the PA-IMO5 treatment compared to the control and other treatments (*p* < 0.05). However, these indices were significantly lower in the IMO10 treatment (containing 10 g/kg IMO) compared to the other groups (*p* < 0.05). The feed conversion ratio in the PA-IMO5 treatment was significantly lower than that in the other groups (*p* < 0.05), but in the IMO10 treatment, it was significantly higher than that in the other groups (*p* < 0.05).

### 3.2. Serum Biochemical Parameters

Serum biochemical parameters of fish fed with PA and IMO are shown in Table 2. Serum protein and albumin values in the PA-IMO5 treatment were significantly higher than those in the control and other groups (*p* < 0.05). Serum globulin values in the PA and PA-IMO5 treatments were significantly higher than those in the other groups (*p* < 0.05). Serum cholesterol, LDL, and glucose levels were significantly lower in the PA and PA-IMO5 treatments (*p* < 0.05), but there were no significant differences in serum triglycerides and HDL levels between the experimental groups (*p* > 0.05).

### 3.3. Serum and Mucosal Immune Parameters

The highest serum lysozyme activity was seen in the PA-IMO5 treatment (*p* < 0.05, Table 3). The serum C3 value in the PA-IMO5 and PA-IMO10 treatments was significantly higher compared to that in the other groups (*p* < 0.05). The serum C4 value showed a similar pattern as its higher value was seen in the PA-IMO5 and PA-IMO10 treatments, while its lower amount was recorded in the IMO10 group (*p* < 0.05).

The effects of adding PA and IMO to the diet on mucosal immune parameters are summarized in Table 4. The total protein content of mucus was highest in the PA-IMO5 treatment compared to the other experimental groups (*p* < 0.05), while its lowest amount was observed in the control and IMO10 treatments (*p* < 0.05). Mucus total Ig levels were significantly higher in the PA and PA-IMO5 treatments (*p* < 0.05). The highest mucus lysozyme activity was observed in the PA-IMO5 treatment (*p* < 0.05).

### 3.4. Antioxidant Enzyme Activity

Serum antioxidant enzyme activity showed a similar pattern in the experimental groups (Table 5). Their activity was lowest in the PA-IMO5 and PA-IMO10 treatments (*p* < 0.05). The serum MDA content showed a similar pattern, with its lowest value seen in the PA-IMO5 and PA-IMO10 treatments (*p* < 0.05).

## 4. Discussion

Our findings showed that adding 1 g/kg PA and 5 g/kg IMO to the diet (PA-IMO5 treatment) significantly increased the final weight, weight gain, and specific growth rate of common carp compared to the control and other groups, which may be attributed to an elevated digestibility of the prebiotic or the enhancement of colonization of the probiotic [29]. The feed conversion ratio was also significantly lower in the PA-IMO5 treatment than in the other groups. Similarly, the highest growth performance was reported in juvenile common carp fed the combination of PA and raffinose (synbiotic) [5]. However, growth indices were significantly lower in the IMO10 treatment (containing 10 g/kg IMO) compared to the other groups, possibly because of the inappropriate dose of IMO for fermentation by probiotics [17].

There is a lot of research reporting the positive effects of PA alone or in combination with prebiotics on growth performance and nutrition indices in different fish species [7, 30, 31]. European sea bass fed dietary PA as a probiotic had significantly higher growth parameters and villus height compared to the control [32]. Besides, probiotics alter the bacterial community in support of improved water quality and subsequently fish growth performance [33]. Moreover, Romano et al. [13] reported that the FCR tended to be lower for juvenile African catfish fed the pregelatinized starch and pregelatinized starch plus isomaltooligosaccharide diets. Dietary pregelatinized starch and isomaltooligosaccharide may enhance available energy for the fish [13]. The positive impact of dietary IMO on fish growth performance is related to its gut mucosa protective function and better nutrient absorption [7]. Prebiotics increase the population of beneficial intestinal bacteria, especially *Bifidobacterium* and lactic acid bacteria. Due to the fermentative condition and acid production in the host intestine, intestinal pH decreases, and therefore, the activity of harmful and pathogenic bacteria is inhibited and the uptake of minerals in the lumen increases [12]. Our results showed that the combination of probiotics (PA) and prebiotics (IMO) as a synbiotic led to an improvement of the growth performance of common carp. This improvement may be due to the effects of probiotic and prebiotic compounds in modifying the intestinal microbial flora [34]. Beneficial bacteria of the gastrointestinal tract have been shown to facilitate the process of digestion and absorption of food and increase its nutritional value, as well as reduce energy consumption by producing enzymes and metabolic substances [35]. The positive effects of synbiotic compounds that have been reported in rainbow trout [36], Japanese flounder [37], common carp [5], and European sea bass [7] support our findings.

A strong innate immune response is related to increasing values of proteins, such as albumin and globulin, which represent the major proteins in serum [38]. In our study, serum protein and albumin values in the PA-IMO5 treatment were significantly higher than those in the control and other groups. Also, serum globulin values in the PA and PA-IMO5 treatments were significantly higher than those in the other groups, indicating that PA or PA in combination with 5 g/kg IMO diets can act as immunostimulants [14, 16].

In this study, a hypocholesterolemia effect was seen after feeding PA or PA-IMO5 diet and the lowest serum cholesterol and LDL levels were recorded in the PA or synbiotic (PA-IMO5) group. These findings confirmed that dietary PA or PA in combination with 5 g/kg IMO can improve the lipid metabolism of juvenile common carp. Serum triglycerides and HDL values were not affected by the experimental diets. Data on the effects of synbiotics on lipid metabolism in fish are contradictory. In Japanese flounder (*Paralichthys olivaceus*), dietary probiotics, prebiotics, and synbiotics decreased triglycerides and LDL but had no effect on cholesterol and HDL [37]. Triglyceride-lowering effects of dietary *Pediococcus pentosaceus* were also reported in common carp, while cholesterol and glucose values did not change following the application of *P. pentosaceus* compared to the control treatment [39]. Moreover, serum glucose levels were significantly lower in the PA and PA-IMO5 treatments. Thus, the PA and PA-IMO5 diets can prevent the increase in glucose values caused by stress.

In the present study, the highest lysozyme activity in serum and mucus was observed in the PA-IMO5 treatment. The serum C3 value in the synbiotic treatments was highest. The serum C4 value showed a similar pattern as its higher value was seen in the synbiotic treatments, while its lower amount was recorded in the IMO10 group. The total protein content of mucus was also highest in the PA-IMO5 treatment compared to the other experimental groups, while its lowest amount was observed in the control and IMO10 treatments. In shrimp (*Litopenaeus vannamei*), while the impact of 0.2% isomaltooligosaccharide individually was negligible, 0.2% isomaltooligosaccharide in combination with 10^8^ CFU/g diet *Bacillus* as a probiotic had significantly positive effects on immune parameters and disease resistance [14]. In the study of Modanloo et al. [6], single or combined administration of galactooligosaccharide and PA significantly enhanced mucus lysozyme activity in common carp fingerlings, with the highest elevation recorded in the probiotic and synbiotic groups. Mucus total Ig levels were significantly higher in the PA and PA-IMO5 treatments in our study. In the study of Hoseinifar et al. [5], probiotics (PA) or PA in combination with 2 g raffinose/kg diet significantly enhanced skin mucus total Ig and protein values compared to the control and prebiotic groups. According to Mohammadi et al. [40], dietary pistachio hull-derived polysaccharide and *P. acidilactici* improved skin mucus and blood immune responses in Nile tilapia. It could be concluded that PA improves the absorption of food in the digestive system [41], which is due to the richness and high diversity of gastrointestinal tract microbial flora, resulting in improved immune system function [42]. In our study, the enhanced immune responses may be related to higher immune ability and general health following dietary supplementation with PA and IMO. It is reported that synbiotics can stimulate immune responses by short-chain fatty acids, which may play a role as an energy source for intestinal epithelial cells and also as a messenger between gut microbiota and immune function through transcriptional pathways [43]. Besides, prebiotics and probiotics may directly induce the bacterial activity of skin lymphoid tissues, modulate the transcription of immune genes, and defend the epidermal skin cells during the pathogenic challenge [44, 45].

Normally, reactive oxygen species (ROS) produced during aerobic metabolism can destroy the cell walls and tissues of the organisms and lead to the weakness or death of the organisms [46]. A positive relationship between health status and antioxidant defense of organisms had been reported [47]. CAT, SOD, and GPx are important antioxidant enzymes that can remove excessive free radicals, reducing the toxicity of ROS. Serum CAT, SOD, and GPx activities were lowest in the synbiotic treatments in our study. However, despite the lower values of antioxidant enzymes, serum MDA contents were also significantly decreased in the synbiotic treatments, suggesting that synbiotic diets can decrease lipid peroxidation and improve the oxidative status in common carp. Hoseinifar et al. [48] had reported similar results in rainbow trout fed galactooligosaccharide and PA. A positive aspect of using probiotics in diets is the improvement of antioxidant defense mechanisms [49]. Also, dietary PA improved the antioxidant defense of *Litopenaeus stylirostris* during the *Vibrio nigripulchritudo* challenge [50]. In rainbow trout fed the prebiotic, probiotic, and synbiotic diets, an increase in antioxidant enzyme activity had been linked to the effects of these dietary supplements on the translation process and/or posttranslational process of this antioxidant enzyme gene expression [51].

In conclusion, our study demonstrated that the combined use of 1 g/kg (10^10^ CFU/kg) *P. acidilactici* and 5 g/kg isomaltooligosaccharide in the diet improved growth performance, feed utilization, blood biochemical parameters, serum (lysozyme, complements C3 and C4) and mucosal (protein, total Ig, and lysozyme) immune responses, and antioxidant defense in fish. Therefore, the combined administration of 1 g/kg (10^10^ CFU/kg) PA and 5 g/kg IMO can be recommended as a beneficial synbiotic additive and immunostimulant in juvenile common carp. In future studies, a similar experiment should be supplemented by a challenge test. Detection of intestinal flora and histological observations can also be suggested.

## Figures and Tables

**Table 1:** Growth parameters of common carp fed different levels of *Pediococcus acidilactici* (PA) and isomaltooligosaccharide (IMO) after 8 weeks.

	Experimental diets					
	Control	PA	IMO5	IMO10	PA-IMO5	PA-IMO10
Initial average weight (g)	17.14 ± 0.74^ns^	17.20 ± 0.82	17.13 ± 0.13	17.25 ± 0.72	17.30 ± 0.70	17.33 ± 0.78
Final average weight (g)	32.69 ± 0.95^b^	35.08 ± 0.95^c^	33.59 ± 0.67^bc^	29.91 ± 0.70^a^	37.37 ± 1.14^d^	32.90 ± 0.82^b^
WG (%)	90.83 ± 6.96^bc^	104.10 ± 4.39^cd^	96.26 ± 5.37^bc^	73.50 ± 4.57^a^	116.40 ± 9.15^d^	90.33 ± 10.17^b^
SGR (%/day)	1.15 ± 0.06^b^	1.27 ± 0.04^bc^	1.20 ± 0.05^b^	0.98 ± 0.04^a^	1.38 ± 0.07^c^	1.15 ± 0.09^b^
FCR	1.92 ± 0.05^ab^	1.78 ± 0.13^ab^	1.87 ± 0.18^ab^	2.24 ± 0.10^c^	1.71 ± 0.06^a^	1.98 ± 0.12^b^
Survival (%)	97.33 ± 2.30^ns^	98.66 ± 1.33	96.00 ± 4.00	96.00 ± 6.92	98.66 ± 2.30	94.66 ± 2.30

Data expressed as mean ± SE. Different letters in each row indicate a significant difference between the experimental groups (*p* < 0.05, *n* = 3). WG: weight gain; SGR: specific growth rate; FCR: feed conversion ratio.

**Table 2:** Blood biochemical parameters of common carp fed different levels of *Pediococcus acidilactici* (PA) and isomaltooligosaccharide (IMO) after 8 weeks.

	Experimental diets					
	Control	PA	IMO5	IMO10	PA-IMO5	PA-IMO10
Protein (g/dl)	4.35 ± 0.23^ab^	4.65 ± 0.20^bc^	4.45 ± 0.16^ab^	4.21 ± 0.11^a^	4.81 ± 0.16^c^	4.47 ± 0.17^ab^
Albumin (g/dl)	1.28 ± 0.13^ab^	1.35 ± 0.17^ab^	1.26 ± 0.11^a^	1.23 ± 0.05^a^	1.49 ± 0.07^b^	1.25 ± 0.09^a^
Globulin (g/dl)	3.07 ± 0.10^ab^	3.30 ± 0.08^b^	3.19 ± 0.05^ab^	2.98 ± 0.17^a^	3.32 ± 0.23^b^	3.22 ± 0.09^ab^
Albumin/globulin	0.41 ± 0.03^ns^	0.41 ± 0.05	0.39 ± 0.03	0.41 ± 0.04	0.45 ± 0.05	0.39 ± 0.02
Cholesterol (mg/dl)	144.2 ± 16.4^ab^	136.8 ± 13.3^a^	149.7 ± 17.5^abc^	164.1 ± 14.7^bc^	136.1 ± 11.3^a^	171.5 ± 8.8^c^
Triglycerides (mg/dl)	279.2 ± 40.9^ns^	258.2 ± 7.3	292.8 ± 12.9	329.1 ± 47.6	255.5 ± 41.04	319.8 ± 56.2
HDL	27.96 ± 2.40^ns^	30.13 ± 2.85	28.08 ± 1.62	27.16 ± 1.72	31.85 ± 3.70	27.38 ± 2.93
LDL	32.40 ± 3.45^b^	17.48 ± 1.86^a^	30.90 ± 3.97^b^	34.87 ± 2.96^b^	15.49 ± 1.48^a^	27.00 ± 7.87^b^
Glucose (mg/dl)	74.83 ± 3.07^b^	62.77 ± 2.12^a^	74.13 ± 3.66^b^	77.36 ± 3.68^b^	63.78 ± 4.66^a^	75.41 ± 4.06^b^

Data expressed as mean ± SE. Different letters in each row indicate a significant difference between the experimental groups (*p* < 0.05, *n* = 3). HDL: high-density lipoproteins; LDL: low-density lipoproteins.

**Table 3:** Blood immune parameters of common carp fed different levels of *Pediococcus acidilactici* (PA) and isomaltooligosaccharide (IMO) after 8 weeks.

	Experimental diets					
	Control	PA	IMO5	IMO10	PA-IMO5	PA-IMO10
Lysozyme (units/ml)	18.67 ± 0.62^b^	21.73 ± 0.51^c^	18.64 ± 0.61^b^	17.50 ± 0.53^a^	23.52 ± 0.57^d^	18.48 ± 0.47^ab^
Complement C3 (mg/dl)	8.21 ± 0.15^a^	8.34 ± 0.08^a^	8.23 ± 0.24^a^	8.03 ± 0.17^a^	9.29 ± 0.25^b^	9.31 ± 0.14^b^
Complement C4 (mg/dl)	2.33 ± 0.14^ab^	2.46 ± 0.13^bc^	2.28 ± 0.17^ab^	2.08 ± 0.07^a^	2.68 ± 0.10^c^	2.60 ± 0.17^c^

Data expressed as mean ± SE. Different letters in each row indicate a significant difference between the experimental groups (*p* < 0.05, *n* = 3).

**Table 4:** Mucosal immune parameters of common carp fed different levels of *Pediococcus acidilactici* (PA) and isomaltooligosaccharide (IMO) after 8 weeks.

	Experimental diets					
	Control	PA	IMO5	IMO10	PA-IMO5	PA-IMO10
Protein (mg/ml)	12.44 ± 0.17^a^	20.28 ± 0.18^c^	16.58 ± 0.50^b^	12.53 ± 0.34^a^	23.54 ± 0.43^d^	16.53 ± 0.46^b^
Total Ig (mg/ml)	4.64 ± 0.51^a^	9.73 ± 0.33^d^	7.51 ± 0.42^c^	5.32 ± 0.29^a^	10.14 ± 0.30^d^	6.45 ± 0.47^b^
Lysozyme activity (U/ml)	8.40 ± 0.35^a^	9.53 ± 0.44^b^	8.47 ± 0.31^a^	8.38 ± 0.35^a^	10.54 ± 0.40^c^	9.23 ± 0.18^b^

Data expressed as mean ± SE. Different letters in each row indicate a significant difference between the experimental groups (*p* < 0.05, *n* = 3). Total Ig: total immunoglobulin.

**Table 5:** Blood antioxidant enzyme activity and MDA value of common carp fed different levels of *Pediococcus acidilactici* (PA) and isomaltooligosaccharide (IMO) after 8 weeks.

	Experimental diets					
	Control	PA	IMO5	IMO10	PA-IMO5	PA-IMO10
CAT (units/ml)	4.29 ± 0.27^b^	4.16 ± 0.41^b^	4.25 ± 0.38^b^	3.52 ± 0.32^b^	3.09 ± 0.18^a^	3.13 ± 0.25^a^
SOD (units/ml)	32.31 ± 2.35^b^	32.15 ± 3.15^b^	33.64 ± 2.81^b^	34.51 ± 3.03^b^	25.49 ± 1.76^a^	27.09 ± 2.25^a^
GPx (munits/ml)	25.39 ± 2.68^b^	26.71 ± 3.17^b^	25.63 ± 2.54^b^	27.48 ± 2.91^b^	21.55 ± 2.27^a^	20.59 ± 2.37^a^
MDA (mM/ml)	95.15 ± 6.82^b^	96.23 ± 7.41^b^	99.71 ± 7.19^b^	97.96 ± 8.34^b^	88.66 ± 6.22^a^	86.27 ± 6.51^a^

Data expressed as mean ± SE. Different letters in each row indicate a significant difference between the experimental groups (*p* < 0.05, *n* = 3). CAT: catalase; SOD: superoxide dismutase; GPx: glutathione peroxidase; MDA: malondialdehyde.

## Data Availability

There is no data available for sharing.
